# Purification and antioxidant properties of triterpenic acids from blackened jujube (Ziziphus jujuba Mill.) by macroporous resins

**DOI:** 10.1002/fsn3.2464

**Published:** 2021-07-23

**Authors:** Yaling Fu, Yanlei Zhang, Rentang Zhang

**Affiliations:** ^1^ College of Food Science and Engineering Shandong Agricultural University Tai’an China

**Keywords:** antioxidant activity, *Blackened jujub*e, purification, triterpenic acids

## Abstract

In order to investigate the purification process and antioxidant activity of triterpenic acids from blackened jujube, the macroporous resin was applied to purify the crude extract from blackened jujube. The adsorption and desorption characterizations of five different macroporous adsorption resins (AB‐8, D‐101, X‐5, HPD‐100, S‐8) for triterpenic acids of blackened jujube were compared, the optimum purification resins were screened, and the purification parameters were optimized. The antioxidant activity of crude extracts and purified products from blackened jujube was analyzed. The results showed that D‐101 resin possessed the best effect on the purification of blackened jujube triterpenic acids. The optimum purification parameters were as follows: sample concentration 25.5 μg/ml, 130 ml of the sample volume was with a flow rate of 2.0 ml/min, eluted with 95% ethanol, and speed flow was 1.0 ml/min. The purity of triterpenic acids was increased by 2.49 times after purification with a recovery rate of (78.58 ± 0.67)%. Furthermore, the IC_50_ values of hydroxyl radical scavenging capacity from triterpenic acids crude extract and purified substances were 0.900 and 0.850 mg/ml, respectively, and the IC_50_ values of superoxide anion radical were 0.745 and 0.594 mg/ml, respectively, indicating that the antioxidative capacity of the purified product was stronger than the crude extract. The purified triterpenic acids (PTA) groups at different doses had excellent protective effects on H_2_O_2_‐induced damage HUVEC cells. Results have revealed that triterpenic acids of blackened jujube have good antioxidant function and utilization and development prospects.

## Graphical abstract

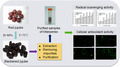

## 1 INTRODUCTION

Jujube (*Ziziphus jujuba* Mill.), belonging to the family of Rhamnaceae, is indigenous to China with a cultivation history of more than 4,000 years and is widespread throughout the temperate and subtropical regions of the Northern hemisphere, especially in North Africa, East Asia, and Middle Eastern countries (Gao et al., 2013; Guo et al., 2015; Rashwan et al., 2020). To date, China is the largest producing and consuming country of jujube in the world, with a production of over 7 million tons annually (Cai et al., 2020). Jujube fruit is well known for its high nutritional value and not only contains trace elements such as calcium, iron, and potassium but also contains a variety of bioactive components, including polysaccharides, phenolics, flavonoids, triterpene acids, and cyclic nucleotides. Due to its multiple healthy functions, such as sedative and hepatoprotective effects, improve sleep quality, and extend the life‐span, the jujube fruit has been commonly applied in health food, medicine herb, and other fields (Ji et al., 2017, 2020). Blackened jujube is a new product of jujube processing. It is formed by controlling the temperature and humidity of the jujube after a period of high‐temperature ripening (Gao et al., 2019). Compared with the original jujube, the color, aroma, flavor, and nutritional components of blackened jujube changed during processing, and the contents of reducing sugar, triterpenoid acids, phenols and flavonoids increased (Sun et al., 2019). Therefore, the research on the active components of blackened jujube can not only increase the economic benefits of jujube but also provide technical support for the development and utilization of its products.

Triterpenoids dominate the bioactive components of jujube fruit, which are widely distributed in the plant kingdom (Song et al., 2020), mostly in the form of free state or ester‐forming glycosides. Studies have indicated that triterpenic acids with antioxidative (Qiao et al., 2014), anti‐inflammatory and antimicrobial (Yu et al., 2012), antitumor (Masullo et al., 2015), hepatoprotective (Zhao et al., 2019), and hypoglycemic activities (Khanra et al., 2017), combined with low toxicity, which has emerged as one of the significant focuses of research in domestic and foreign. For example, betulinic acid isolated from jujube fruit inhibited the growth of human breast cancer MCF‐7 cells (Sun et al., 2013). Triterpenes 9 and 10 were proved to be strong free radical scavenging activities in sour jujube (Qiao et al., 2014). However, the crude triterpenoid acid extract in jujube usually contains protein, polysaccharide, and other impurities, which affected the study of its physiological activity. Therefore, a high‐efficient separation and purification technology were needed to acquire triterpenoids with high purity. Macroporous adsorption resins have been extensively used as the separation and purification of natural products, attributing to their excellent chemical stability and adsorption selectivity, large surface area, renewable utilization, and insoluble in acid, alkali, and organic solvents (Wu et al., 2018; Xi et al., 2015). Researches like pentacyclic triterpenes from Centella asiatica leaves (Yingngam et al., 2020), total flavonoids from Pteris ensiformis Burm. (Hou et al., 2020), and cocoa polyphenols from cocoa bean husks (Zhong et al., 2019) have been reported.

In vitro antioxidant activities of plants triterpenoids mainly focus on radical scavenging antioxidant assessment, while few studies focus on cell level antioxidant evaluation. The results showed that H_2_O_2_ had become a commonly used reagent in the construction of cell oxidative pressure model (Bhakkiyalakshmi et al., 2014), and the model cells induced by H_2_O_2_ can more accurately reflect the antioxidant effect of active ingredients. However, to date, no studies have focused on the cellular antioxidant activity of triterpenic acids from blackened jujube.

Therefore, in this research, five macroporous resins were used to enrich triterpenic acids from the blackened jujube. The resin screening and adsorption kinetics were first investigated, and then, the purification parameters were optimized through static and dynamic adsorption and desorption experiments. Additionally, to better utilize the triterpenoid acids from blackened jujube, the antioxidant activity of the triterpenoids‐purified extracts and crude extracts was analyzed by radical scavenging experiments. Then, to further explore antioxidant activity, the protective effects of purification products on H_2_O_2_‐induced HUVEC cells were investigated. This work would demonstrate that it is worth studying the biological activity of triterpenoids from blackened jujube and provide the basis for the development and utilization of functional foods and pharmaceuticals.

## 2 MATERIALS AND METHODS

### 2.1 Materials and reagents

The raw material of jujube was produced from Xinjiang, China, and was purchased from Tai'an Market. The jujube with neat fruit shape was first soaked in water for 1 hr, then bagged and sealed, and then processed in a drying oven with a temperature of 75°C and a humidity of 80% for about 55 hr to produce blackened jujube, and the blackened jujube with a water content of about 26%, a color of dark brown with a sweet and sour taste. After blackening, they were cut into a fine flap, dried in the oven at 60°C, then ground to fine powders and sifted 40 mesh, put into the sealed bag for standby.

Standard oleanolic acid (purity ≥98%, HPLC) was provided by Shanghai Yuanye Biotechnology Co., Ltd. Macroporous resins including HPD‐100, X‐5, S‐8, D‐101, and AB‐8 were acquired from Cangzhou Baoan Technology Co., Ltd. MTT and penicillin–streptomycin (PS) were purchased from Beijing Dingguo Changsheng Biotechnology Co., Ltd. Hoechst 33258 reagents kit was bought from Nanjing Kaiji Biotechnology Co., Ltd. DMEM high glucose, fetal bovine serum (FBS), and dimethyl sulfoxide (DMSO) were purchased from Hyclone Company and Sigma‐Aldrich, respectively. All other reagents were of analytical grade, and all experiments were conducted with deionized water.

### 2.2 Extraction of triterpenic acids

An appropriate amount (100 g) of blackened jujube powder was subjected to triterpenoids extraction with 60% (v/v) ethanol solution at a ratio of 1:20 (w/v) for 45 min twice using ultrasonic assistance. The supernatant was filtered and concentrated by a rotary evaporator at 50°C. After concentration, the obtained crude triterpenoid solutions were added diatomite to remove macromolecular protein and sugar and then defatted with petroleum ether and extraction with ethyl acetate. The extraction solution in the upper layer was concentrated under a partial vacuum and stored at −20°C for further use (Wei et al., 2015).

### 2.3 Determination of triterpenoids content

The content of triterpenoids was monitored based on the vanillin‐perchloric acid assay method (Cai et al., 2019) with slight modification. Briefly, sample solution (0.1 ml) in a water bath to evaporate solvent firstly and then added 0.2 ml vanillin‐acetic reagent (5:95 w/v, currently prepared) and perchloric acid (0.8 ml). The mixture was shaken well and reacted at 60°C for 15 min, followed by 5.0 ml glacial acetic acid. After 10 min, the absorbance value at 574 nm was measured using a UV spectrophotometer (Shanghai Oppel Instrument Co., Ltd.). The regression equation was expressed as *y* = 0.0409*x *− 0.01, (*R* ^2^ = 0.9989) with oleanolic acid as the reference, where *y* was absorbance and *x* was total triterpenoids concentration (μg/ml).

### 2.4 Purification of triterpenes with macroporous resins

#### 2.4.1 Pretreatment of macroporous resins

The resins were first soaked in two times volume 95% (v/v) ethanol for 24 hr to fully swell the resins, filtered out the ethanol, and rinsed with deionized water until it had no ethanol flavor. Then, the washed resins were immersed with 5% HCl and 5% NaOH for 4 hr, respectively. Lastly, they were washed with deionized water until the pH of filtrate reached a neutral and soaked in deionized water at room temperature for standby (Bao et al., 2015).

#### 2.4.2 Static adsorption and desorption experiments

##### Screening of macroporous resins

5.0 g of weighed prepared resins were placed in a 100 ml Erlenmeyer flask with a stopper, and 40 ml crude triterpenic acids solution was added. The flasks were continually shaken for 12 hr at 120 rpm and 25°C to reach the adsorption equilibrium, filtered, and took the filtrate to determine the concentration of triterpenic acids. Subsequently, the remaining resin was washed with deionized water until eluents were colorless, followed by desorption with 40 ml of 95% (v/v) ethanol, which was shaken (120 rpm) in a thermostated vibrator (Changzhou Kaihang Instrument Co., Ltd.) at 25°C for 24 hr. After desorption, the triterpenic acids contents of the supernatant were measured. The adsorption/desorption capacity and the ratio of each resin were calculated with the formulas below.

(1) $$Q_{e}=\frac{\left(C_{o}‐C_{e}\right)V_{1}}{W}$$

(2) $$A=\frac{C_{o}‐C_{e}}{C_{o}}\times 100\%$$

(3) $$Q_{d}=\frac{C_{2}V_{2}}{W}$$

(4) $$D=\frac{C_{2}V_{2}}{\left(C_{o}‐C_{e}\right)V_{1}}\times 100$$

where *Q*_e_ and *Q*_d_ are the adsorption and desorption capacity at equilibrium (μg/g), respectively; *C*_o_ and *C*_e_ represent the original and adsorption equilibrium concentrations of triterpenic acids in the solutions (μg/ml), respectively; *C*_2_ is the concentration of triterpenic acids in the desorption solutions (μg/ml); *V*_1_ and *V*_2_ are the initial sample and desorption solutions volume (ml), respectively. *A* and *D* stand for the adsorption and desorption ratio (%); *W* is the dry weight of resin (g).

##### Adsorption kinetics experiment

According to adsorption and desorption capacities, the initially selected D‐101 resin was employed in the adsorption kinetics study. 5.0 g of accurately weighed pretreated resin was conducted in 100 ml conical flasks containing 50 ml of the extract solution. Then, the flask was continually shaken (120 rpm) for 10 hr at 25°C. Supernatants were taken out at specific time intervals till equilibration, and the concentrations of triterpenic acids in the solution were determined to plot the static adsorption kinetics curve.

#### 2.4.3 Dynamic adsorption and desorption experiments

The dynamic experiments of adsorption and desorption were performed in a chromatographic column (300 × 16 mm) wet‐packed with the D‐101 resin as selected. The crude triterpenic acids solution of blackened jujube was filtered through a 0.45 μm membrane firstly and then applied to the glass columns at a suitable flow rate. A tube of effluent was collected every 10 ml to measure the triterpenic acid concentration of the effluent. The dynamic leakage curve of resin was drawn to determine the best feed volume. In order to study the influence of various flow rates and sample concentrations on dynamic adsorption, sample solutions containing different concentrations (10.5, 15.5, 20.5, 25.5, and 30.5 μg/ml) were loaded onto the column at the flow rates (1, 2, 3, and 5 ml/min). The effluent liquid was collected for detecting the triterpenic acids contents with UV spectrophotometry.

After reaching adsorption equilibrium, the deionized water was first used for washing the resin column and then eluted with stepwise gradient ethanol solution (40%–100%, v/v) at a flow rate of 1.0 ml/min for the desorption process. The desorption results were analyzed at different ethanol concentrations. Subsequently, the optimized desorption solution was used to elute resin at gradient flow rates (1, 2, 3, 4, and 5 ml/min) to investigate the effect of desorption flow rate. The triterpenoid acids eluents were collected and monitored by the UV spectrophotometry method.

Moreover, the elution curve was drawn as follows: the optimal loading conditions and elution processes were set on the basis of the above experiments of dynamic adsorption and desorption, then the elution was performed under the optimized conditions, the eluent was collected at 5 ml intervals, and the triterpenic acids concentration in each desorbed part was measured using UV spectrophotometry to plot the dynamic elution curve.

#### 2.4.4 Preparative purification with optimal conditions

According to the optimized purification conditions, D‐101 resin was applied for dynamic adsorption and desorption of crude triterpenic acids solutions. After the eluant was collected and concentrated by a rotary evaporator at 50°C, followed by frozen‐dried, the dried products were weighed and dispersed in 80% ethanol to obtain sample solutions. The sample solution concentration was measured, and the formulas below were used for calculating triterpenic acids purity and recovery rate.

(5) $$P\left(\%\right)=\frac{m}{M}\times 100$$

(6) $$Q\left(\%\right)=\frac{C_{2}\times V_{2}}{C_{1}\times V_{1}}\times 100$$

where *P* is the triterpenic acids purification level (%); *m* is the triterpenic acids content of the sample solution (mg); *M* is the sample weight (g); *C*_1_ is the concentration of prepurified triterpenic acids solution (μg/ml); *V*_1_ is the total volume of prepurified triterpenic acids solution (ml); *C*_2_ is the concentration of purified triterpenic acids solution (μg/ml); and *V*_2_ is the total volume of purified triterpenic acids solution (ml).

### 2.5 In vitro antioxidant activity analysis

#### 2.5.1 Radical scavenging activity

Purified triterpenic acids, crude extract, and *V*_C_ were prepared into different concentrations of solutions to be tested and used. The hydroxyl radical scavenging activity was estimated according to the reference procedure of Qiu et al. (2019), with appropriate modifications, and the scavenging activity of superoxide anion was analyzed as described by Wang et al. (2018).

### 2.6 Cellular antioxidant activity

#### 2.6.1 Cell culture

Human umbilical vein endothelial cells (HUVECs) were provided by the Cell Bank of Type Culture Collection of the Chinese Academy of Sciences (Shanghai, China). Penicillin–streptomycin and fetal bovine serum were placed in a 37°C incubator for complete melting. Under aseptic conditions, HUVECs cells were cultured in DMEM high glucose medium mixed with antibiotics (penicillin and streptomycin) and fetal bovine serum (1:100 and 1:10 volume ratio) in an incubator at 37°C and containing 5% CO_2_.

#### 2.6.2 MTT assay

MTT test was used on the cell viability analysis according to the results published in the literature (Zhu et al., 2017). HUVECs cells were inoculated in 96‐well plates (8 × 10^3^ cells/well) for incubation 24 hr, and then, cells were exposed to indicated concentrations of H_2_O_2_ (100–600 μM) for 24 hr stimulation. Moreover, cells were incubated using PTA (0.5, 1.0, and 2.0 mg/ml), and H_2_O_2_ (300 μM ) for 24 hr, followed by 20 μl MTT (0.5 mg/ml), was dropped in each well at 37°C for 4 hr incubation. Subsequently, the medium solution in each well was replaced with 150 μl DMSO and incubated for 10 min. The optical density at 490 nm was measured utilizing a microplate reader (Seymour Fisher Instruments Co., Ltd).

#### 2.6.3 Hoechst 33258 fluorescence staining

The amount of cell apoptosis was detected by Hoechst 33258 fluorescence staining. HUVEC cells were transferred into 12‐well plates (8 × 10^4^ cells/well) and treated with 300 μM H_2_O_2_ and different concentrations (0.5, 1.0, and 2.0 mg/ml) of the PTA, after 24 hr incubation, operated according to Hoechst 33258 kit, and then the anti‐fluorescence quenching agent was added for sealing tablets treatment, and the images were obtained under fluorescence microscope (Nikon Instrument Co., Ltd) with excitation wavelength of 340 nm.

#### 2.6.4 MMP assay

Fluorescent probe Rhodamine 123 (Nanjing Kaiji Biotechnology Co. Ltd) was applied to estimate mitochondrial membrane potential as reported by Chu et al. (2019). HUVEC cells were inoculated in the cell coverslips of 12‐well plates (8 × 10^4^ cells/well). After adhering to the wall, the drug was added for incubation 24 hr. Subsequently, the old medium was sucked out and washed two times with PBS. 500 µl rhodamine 123 solutions (10 µg/ml) was subjected to each well and further cultured at 37°C for 20 min in an incubator. After the unloaded rhodamine 123 solution was washed twice with PBS, the cells were fixed with 4% paraformaldehyde solution for 30 min at room temperature. Anti‐fluorescence quenching agent was added for sealing tablets, and the images were acquired by fluorescence microscope.

#### 2.6.5 Measurement of ROS levels

A 2',7'‐dichlorofluorescein diacetate (DCFH‐DA) assay kit (Jiancheng Institute of Biotechnology, Nanjing, China) was conducted to assay the reactive oxygen species referring to previous research (Zhao et al., 2018). Briefly, HUVEC cells were planted in 12‐well plates (8 × 10^4^ cells/well) and cultured with 300 μM H_2_O_2_ and different concentrations of PTA (0.5, 1.0, or 2.0 mg/ml) for 24 hr. After incubation, the final concentration of 500 μl DCFH‐DA (10 μmol/L) was added and incubated for 45 min at 37°C with 5% CO_2_ avoiding light. After washing gently three times with PBS and fluorescent microscope was used to examine the fluorescent.

### 2.7 Statistical analysis

All experiments were repeated three times, and the data expressed as mean value ± standard deviation (*SD*). The significance of differences was assessed using one‐way analysis of variance (ANOVA) combined with Duncan' test by SPSS 20.0 software at *p* < .05 and *p* < .01.

## 3 RESULTS AND DISCUSSION

### 3.1 Research on purification process

3.1

#### 3.1.1 Screening of macroporous resins for the purification of triterpenic acids

The adsorption of macroporous resins depends on van der Waals force and hydrogen bonding between adsorbate and adsorbent, and the interaction between them is a physical process. Besides, chemical structures, polarities, surface area, and pore diameter are also the indicators that affect the adsorption and desorption of resins (Yang et al., 2016; Yu et al., 2020). Hence, in order to enrich blackened jujube extracts, five kinds of macroporous resins were used as screen in the same experimental conditions. The physicochemical properties, static adsorption, and desorption results of various resins were displayed in Table 1. The results suggested that D‐101 resins have better adsorption and desorbing effects on triterpenic acids of blackened jujube compared with other types of resins, which might be due to the fact that triterpenoids were weakly polar substances, while D‐101 resins were nonpolar and have good interaction with them. Meanwhile, it might also be related to its larger specific surface area and pore size. Therefore, according to the adsorption and desorption of resins, D‐101 was employed as the suitable resin for the following experiments. D‐101 resin was selected as the optimal resin for subsequent study.

**TABLE 1:** Physicochemical parameters, adsorption/desorption capacity, and ratio of triterpenoids on different resins

Resin type	Surface area (m^2^/g)	Polarity	Pore size A°	Adsorption capacity (μg/g)	Adsorption rate (%)	Desorption capacity (μg/g)	Desorption rate (%)
AB‐8	480 ~ 520	Weak polar	130–140	86.91 ± 3.36 ^c^	37 ± 1.43 ^b^	57.36 ± 3.17 ^c^	65 ± 2.03 ^b^
D‐101	550 ~ 600	Nonpolar	90–100	117.73 ± 1.67 ^a^	50 ± 0.71 ^a^	85.15 ± 1.66 ^a^	73 ± 2.45 ^a^
X‐5	500 ~ 550	Nonpolar	290–300	108.73 ± 1.46 ^b^	47 ± 3.11 ^a^	73.95 ± 3.05 ^b^	68 ± 1.48 ^b^
HPD‐100	650 ~ 700	Non polar	85–90	87.57 ± 2.65 ^d^	34 ± 1.29 ^bc^	46.21 ± 4.54 ^d^	60 ± 1.48 ^c^
S‐8	100 ~ 120	Polar	280–300	77.12 ± 3.20 ^d^	33 ± 2.90 ^c^	42.85 ± 2.20 ^d^	56 ± 0.87 ^d^

Note

Different superscripts in the same column indicate significant difference (*p* < .05).

#### 3.1.2 Adsorption kinetics of D‐101 resin

**FIGURE 1:**
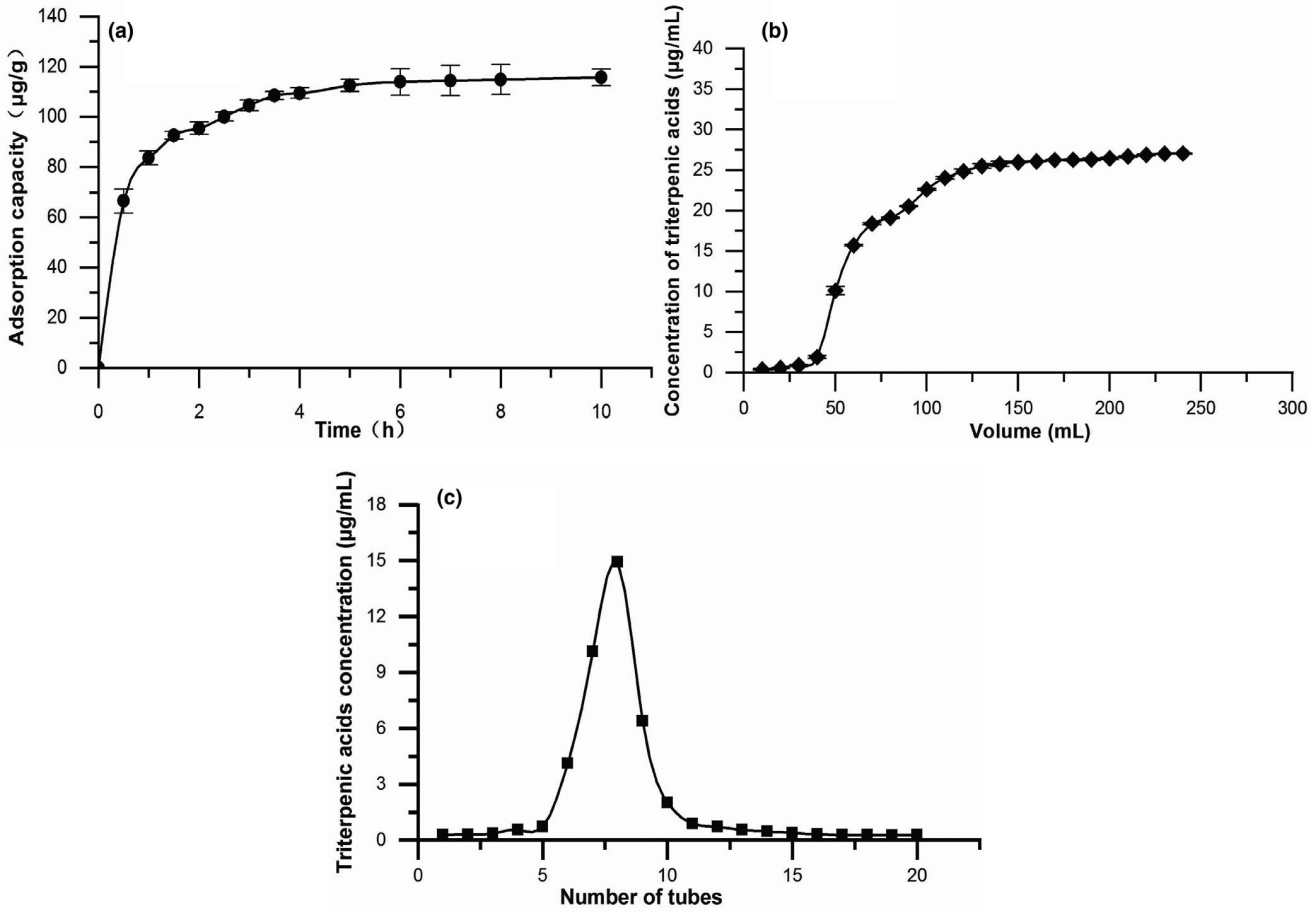
Adsorption and desorption behavior of triterpenic acids of blackened jujube on D‐101 resin. (a) Static adsorption kinetic curve. (b) Dynamic leakage curve. (c) Dynamic elution curve at the optimal parameters

The adsorption kinetics of D‐101 resin for triterpenic acids of blackened jujube was shown in Figure 1a. The unit adsorption capacity changed greatly within the first 1.5 hr. After 5 hr, the adsorption amount changed slowly and tended to be stable basically, indicating that the resins reached the adsorption saturation state. The adsorption rate was faster in the initial stage, which possibly ascribed to the D‐101 resin having more adsorption surface area and sorption sites. Subsequently, triterpenic acids molecules increased gradually in the resin, the repulsive force between the molecules on resin surface and the mass transfer resistance within the particles made it more difficult for triterpenoid acid molecules to occupy (Jin et al., 2015), resulting in slow adsorption. Therefore, the adsorption time of 5 hr was appropriate.

#### 3.1.3 Dynamic adsorption and desorption

##### Dynamic leakage curve

In the process of resin adsorption, normally, the leakage point was considered as the concentration of target compound in the effluent reached 1/10 of the initial concentration (Wu et al., 2015). In the leakage curve (Figure 1b), the triterpenic acid concentration of the effluent gradually increased when loading sample volumes increased. At 60 ml, the mass concentration of the effluent was 15.73 μg/ml, which was close to 1/10 of the upper column solution concentration, reaching the leakage point. When the effluent reached 130 ml, the concentration of triterpenic acids in the effluent was 25.23 μg/ml, which was close to that of the sample solution (25.50 μg/ml), indicating that the resin reached adsorption saturation. So, the loading volume was determined to be 130 ml.

##### Effect of concentration of sample solution on the adsorption capacity

**FIGURE 2:**
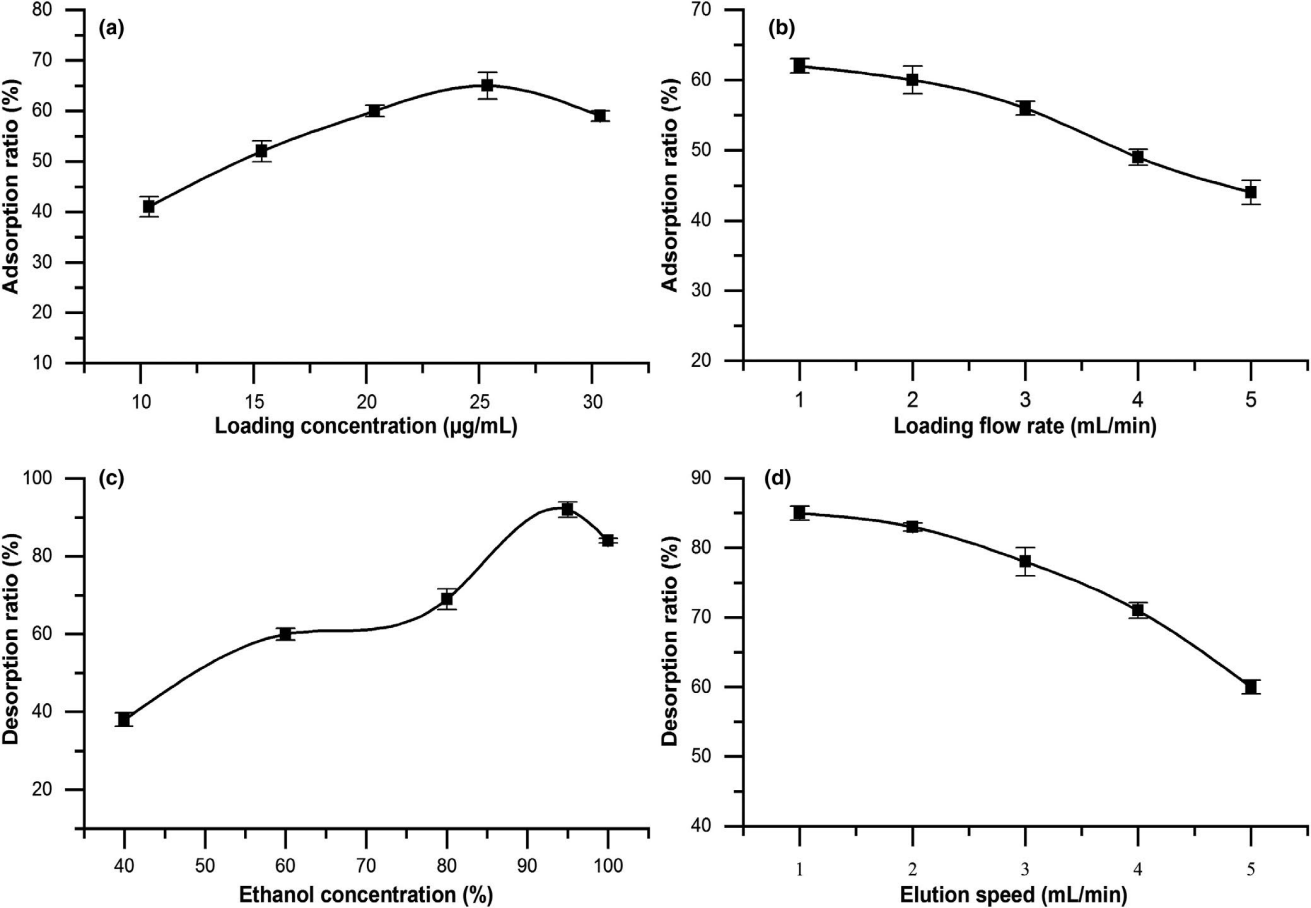
Factors affect the adsorption and desorption properties of D‐101 resin. (a) Effect of sample concentration on adsorption capacity. (b) Effect of flow rate on adsorption capacity. (c) Effect of ethanol concentration on desorption ratio. (d) Effect of flow rate on desorption ratio

As observed in Figure 2a, the adsorption ability for triterpenic acids increased first and then decreased with the increase in sample concentration, which reached a maximum of 25.5 μg/ml sample concentration with 65% adsorption rate of triterpenic acids. This phenomenon might be attributed to the fact that as the concentration increased, the resin had not reached the adsorption saturation, and there was enough space to adsorb triterpene acids in the solution, which led to the adsorption rate rose. However, when sample concentration was too high, the impurities contained also increased and the free active site on the resin reduced; the target substance and impurities compete for the active site, resulting in the adsorption capacity dropped (Sun et al., 2016). Thus, the mass concentration of loading sample was chosen as 25.5 μg/ml.

##### Effect of flow rate on the adsorption capacity

When the sample loaded at different speeds, the contact time between the target and the resin would be different, so the adsorption rate would be different. Figure 2b showed the adsorption ratio reduced for triterpenoic acids gradually as the sample flow rate increased. This reason could be explained by the fact that with the increase in sample flow rate, the contact time between the target object and the resin could be shortened, and the object flowed out of the column before it was adsorbed, resulting in overload leakage and reducing the adsorption effect of the resin (Huang et al., 2017). Therefore, a lower flow rate was conducive to the full adsorption of resin, but in practice, a lower feed flow rate would lead to too long adsorption time and extend the experimental cycle. Finally, the proper flow rate of sample loading was 2 ml/min.

##### Effect of ethanol concentration on desorption ratio

Different concentrations of ethanol were used to establish the proper desorption conditions to select the appropriate desorption ethanol concentration. The desorption rate of target compound was the highest using 95% (v/v) ethanol eluted the resin, and the desorption rate declined when the ethanol concentration was increased or decreased (Figure 2c). This phenomenon might be related to the polarity of triterpenes. Ethanol reduced water polarity, and triterpenes were highly soluble in low‐polar eluent, while the impurities rose in high concentration ethanol (Aalim et al., 2018; Hou et al., 2020). Considering that the elution effect of triterpenes, 95% ethanol was proposed for elution.

##### Effect of elution speed on desorption ratio

The effect of elution speed on desorption ratio was summarized in Figure 2d. The desorption rate of triterpenic acids from blackened jujube decreased as elution flow rate rose. The possible reason for this was that when the elution speed was fast, the contact time between the eluent and the resin was short, the target substance in the resin was not replaced in time, and the desorption was incomplete. Therefore, the higher the elution velocity, the worse the desorption effect. When the elution speed was low, the resin micropores were easier to allow ethanol to enter, and the triterpene molecules were dissolved entirely and eluted (Jia & Lu, 2008). Hence, the triterpenoids were more thoroughly desorbed at lower elution rate. However, if the elution speed was too slow and the desorption time was prolonged, the experimental progress would be affected. Considering comprehensively, the optimal flow rate was determined to be 1.0 ml/min.

##### Dynamic elution curve

Figure 1c showed the elution curve of triterpenes under optimum desorption conditions. The elution was carried out at a rate of 1.0 ml/min with 95% ethanol concentration, and triterpenic acids were basically eluted when the eluent was connected to the fifth tube, the volume of eluent was 25 ml. The absorbance value of triterpenic acids reached a peak at the 7th tube of eluent, and the triterpenic acid compounds were basically eluted when the eluent was collected to the 16th tube. The peak shape and width of the elution peak were sharp and small, and there was no tailing phenomenon. Therefore, 5–9 tubes were selected as eluent of triterpenoid acid, and the amount of eluent was 80 ml.

### 3.2 Determination of triterpenic acids purity in blackened jujube after purification

The validation experiments were carried out in accordance with the process conditions determined above. The 95% ethanol eluent was freeze‐dried and weighed. The result showed that the triterpenic acids of blackened jujube were purified by dynamic adsorption and desorption of D‐101 resin, the content was increased from (23.55 ± 0.60)% to (58.77 ± 0.52)%, which was about 2.49 times than before purification, and the recovery was (78.58 ± 0.67)%. This study confirmed that the method had high efficiency and good separation effect and was suitable for the purification of triterpenic acids in blackened jujube.

### 3.3 Antioxidant activity in vitro

#### 3.3.1 Comparative antioxidant activities assay of crude and purified extract

**FIGURE 3:**
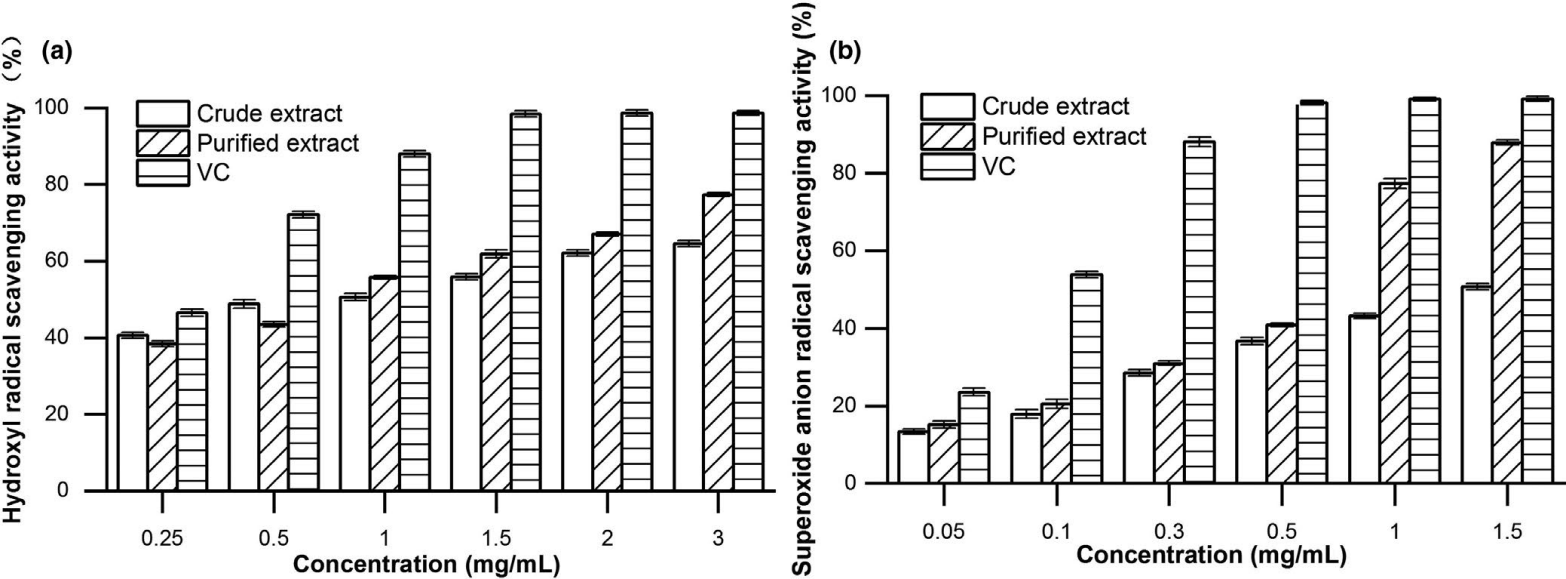
Antioxidant activities of crude and purified triterpenic acids from blackened jujube in vitro. (a) Hydroxyl radical scavenging activity. (b) Superoxide anion radical scavenging activity

Different concentrations of triterpenic acids crude extract and purified product exhibited moderate •OH radical scavenging activity in a dose‐dependent manner, as can be seen in Figure 3a. Before the mass concentration of 0.5 mg/ml, the scavenging effect of crude triterpenoid acids was higher than that of purified product. Increasing the concentration of samples from 1.0 mg/ml to 3.0 mg/ml enhanced the scavenging capacity of the purified triterpenic acids compared with the crude extract. At the concentration of 3.0 mg/ml, the hydroxyl radical scavenging rates of V_C_, triterpenoid acid crude extract, and purified product reached the maximum value of 99.43%, 64.57%, and 77.37%, respectively, which indicated that the scavenging ability of triterpenic acids to •OH was enhanced after purification. Furthermore, the O_2_^−^• scavenging ability of triterpenic acids samples showed an increasing trend with the increase in sample concentration before and after purification. However, at the same mass concentration (1.5 mg/ml), V_C_ was the strongest (99.13%), followed by the purified product (87.93%), and the crude extract was the weakest (50.73%). The growth trend of V_C_ tended to be stable, while the purified triterpenic acids showed a steep upward trend and higher than that of crude extract during 0.5 ~ 1.5 mg/ml (Figure 3b). This result indicated that the purified triterpenic acids had a better scavenging effect on superoxide anion.

#### 3.3.2 The protective effect on H_2_O_2_‐damaged HUVEC cells

##### The PTA inhibits H_2_O_2_‐induced cell cytotoxicity in HUVEC cells

**FIGURE 4:**
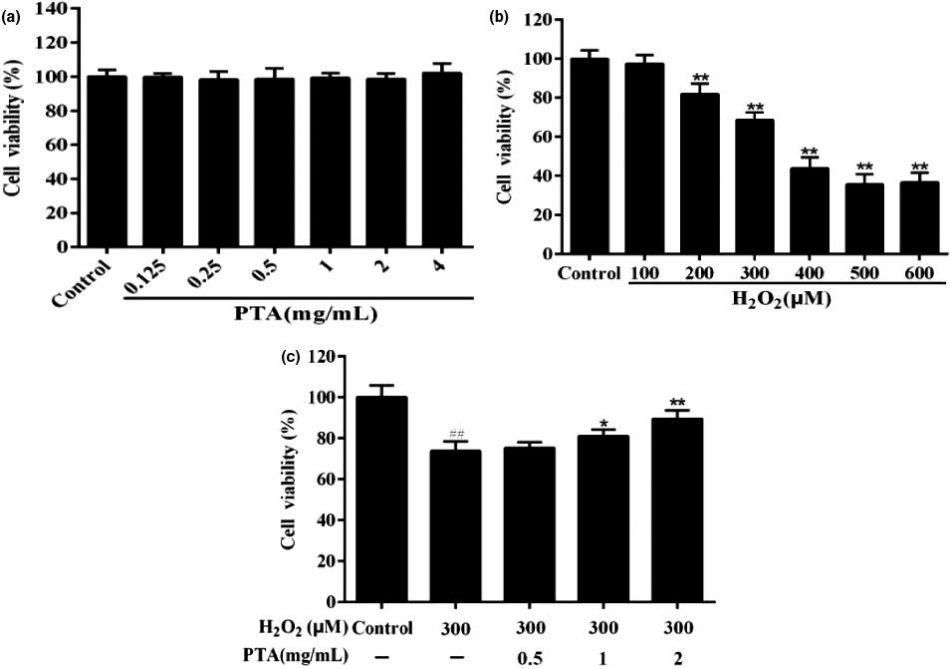
Protective effects of the purified triterpenic acids (PTA) on H2O2‐damaged HUVEC cells. (a) HUVCE cells were treated with different concentrations of Hesp for 24 hr. (b) The HUVEC cells were treated with different concentrations of H2O2 for 24 hr. (c) The cells were subjected to PTA and H2O2 (300 μM) for 24 hr. Cell viability was measured via MTT assays. All data were expressed as mean ± standard deviation. *p < .05 and **p < .01 versus H2O2 group. ##*p* < .01 versus control group

We further evaluated the antioxidant activity of purified triterpenic acids (PTA) in HUVEC cells by H_2_O_2_ induced on the above radical scavenging antioxidant results basis. The MTT assay results revealed that the survival rate of cells did not change significantly at the concentrations of PTA (0.125 ~ 4.0 mg/ml) (Figure 4a). In addition, as shown in Figure 7b, the different administration of H_2_O_2_ resulted in a significant decrease (*p* < .01) in the cell survival rate. Less than 50% reduction in cell viability occurred when the concentration of H_2_O_2_ exceeded 300 μM (68.63% ± 3.89%) and significantly different from that of the control group (*p* < .01). Therefore, the study dose was chosen at 300 μM H_2_O_2_ (Figure 4b). Furthermore, the HUVEC cells were incubated with PTA (0.5, 1.0, and 2.0 mg/ml) and 300 μM H_2_O_2_ for 24 hr, the viability of HUVEC cells presented an increasing trend as the concentration of the PTA rose, which was a significant difference that of the H_2_O_2_ treated group (*p* < .01 or .05). These results suggested that purified triterpenic acids (PTA) inhibited the cell cytotoxicity by H_2_O_2_ damaged (Figure 4c).

##### The PTA decreased the H_2_O_2_‐induced cell apoptosis in the HUVEC cells

**FIGURE 5:**
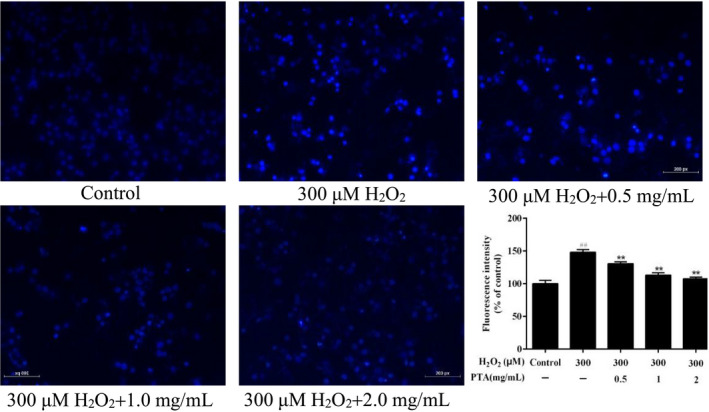
Induction of PTA on apoptosis of HUVEC cells induced by H2O2. The data were represented as the mean ± *SD*. ##*p* < .01 compared with control group, **p < .01 versus group treated with H2O2

The results of HUVEC cell apoptosis detected via Hoechst 33258 staining were demonstrated in Figure 5. In the H_2_O_2_ treatment group, apoptotic bodies were formed, and the number increased. The irregular shape of the nucleus and a significant increase in the bright blue fluorescence occurred (*p* < .01). After the treatment with various concentrations of PTA, the number of nuclear fragments and the blue fluorescence intensity significantly reduced, and there was a dose‐effect relationship (*p* < .01), indicating that PTA could effectively alleviate the apoptosis induced by H_2_O_2_.

##### Effect of the PTA on MMP reduction in HUVEC cells

**FIGURE 6:**
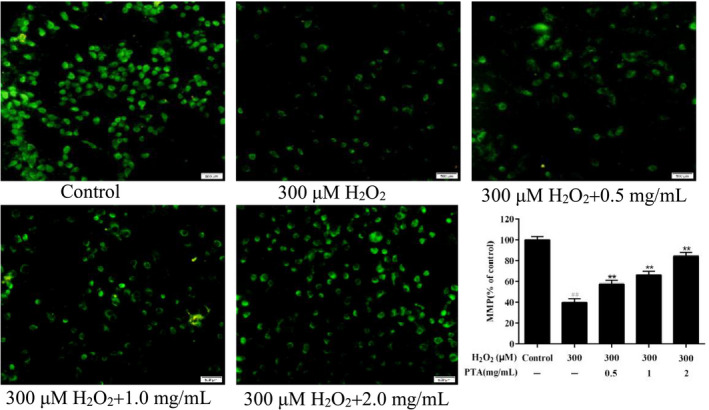
Effect of PTA on the decrease of mitochondria membrane potential level in HUVEC cells induced by H2O2. Data were displayed as the mean ± standard deviation. ##*p* < .01 versus control group, **p < .01 versus group treated with H2O2

Studies have shown that the vital indicator of mitochondrial dysfunction and cell apoptosis was taken for loss of mitochondrial membrane potential (Song et al., 2017). Therefore, the changes in mitochondrial membrane potential in HUVEC cells were detected in this work. As presented in Figure 6, the treatment of H_2_O_2_ reduced significantly cells fluorescence intensity (*p* < .01), which meant that mitochondrial function was destroyed. In contrast, different concentrations of PTA treatment significantly elevated the fluorescence intensity of cells (*p* < .01), showing a concentration‐dependent relationship. The results showed that the PTA could alleviate oxidative stress induced with H_2_O_2_ by inhibiting ROS production and mitochondrial dysfunction.

##### The PTA reduced H_2_O_2_‐induced ROS generation in HUVEC cells

**FIGURE 7:**
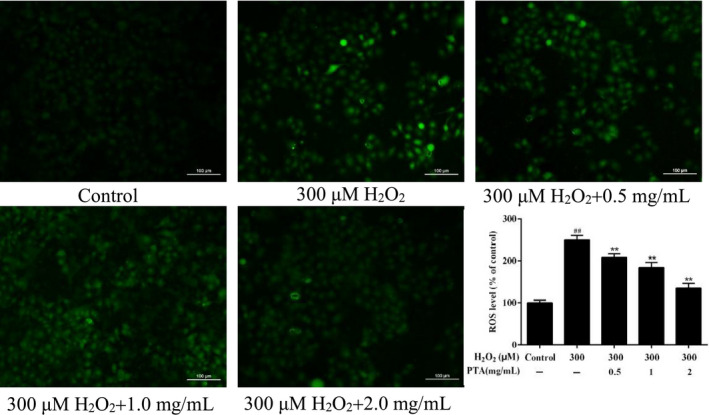
The effect of PTA on the production of ROS level in H2O2 induced HUVEC cells. Values represented as mean ± *SD* of three experiments. *p < .05, **p < .01 versus the group treated with H2O2, and ##*p* < .01 versus the control group

The generation of cellular ROS was adopted to investigate oxidative stress (Cao et al., 2019). After cells were treated using H_2_O_2_ and three concentrations of PTA (0.5, 1.0, and 2.0 mg/ml) for 24 hr, the generation of ROS in HUVEC cells was explored by fluorescent probe DCFH‐DA. As shown (Figure 7), the ROS level in HUVEC cells treated by H_2_O_2_ was significantly rose (*p* < .01), and the fluorescence intensity reached 100.00% ± 6.13%, which was about 2.5 times that of the control group (249.77% ± 11.18%), implying that H_2_O_2_ treatment could promote the increase in intracellular ROS levels. When the PTA was added to protect the H_2_O_2_‐damaged HUVEC cells, the relative intracellular ROS production was declined significantly (*p* < .01) with increasing sample concentration. These results suggested that the PTA provided protection against H_2_O_2_ stress in HUVEC cells by decreasing ROS generation.

## 4 CONCLUSIONS

In this work, the results confirmed the purification method of blackened jujube triterpenoid acid established was practicable. The experiment was carried out to screen macroporous resins, and D‐101 was identified as the best resin for the crude triterpenoid acid enrichment of blackened jujube. Through static and dynamic experiments, the optimum process parameters of resin were obtained. The mass concentration of the sample solution was 25.5 μg/ml, the maximum loading volume was 130 ml, and the loading flow rate was 2.0 ml/min, the amount of eluent was 80 ml, the elution flow rate was 1.0 ml/min, and 95% ethanol solution was used for elution. Under these conditions, the purity of triterpenic acids in blackened jujube was increased from 23.55% to 58.77%, and the recovery was (78.58 ± 0.67%). The proposed method was simple, low cost, and easy in scaling‐up and was suitable for the enrichment and purification of triterpenoids. In vitro antioxidant activity experiment showed that the crude and purified triterpenic acids of blackened jujube had scavenging effects on •OH radical and O_2_^−^• radical, and the antioxidant capacity of triterpenic acids purified by D‐101 resin was significantly enhanced. MTT, Hoechst 33258, and ROS levels showed that the purified triterpenic acids of blackened jujube had a superior protective action on H_2_O_2_ damaged of HUVECs cells. To sum up, this conclusion provides a theoretical basis for the development of natural antioxidants and has a certain guiding significance for the industrial production and application of triterpenes on blackened jujube.

## CONFLICTS OF INTEREST

The authors declare that there are no conflicts of interest.

## AUTHOR CONTRIBUTIONS

**Yaling Fu:** Conceptualization (lead); Writing‐original draft (lead); Writing‐review & editing (lead). **Yanlei Zhang:** Data curation (equal); Formal analysis (equal). **Rentang Zhang:** Funding acquisition (lead); Supervision (lead).

## ETHICAL REVIEW

This study does not involve any human or animal testing.

## INFORMED CONSENT

Written informed consent was obtained from all study participants.

## Data Availability

Research data are not shared.
